# Genomic diversity, chromosomal rearrangements, and interspecies hybridization in the *Ogataea polymorpha* species complex

**DOI:** 10.1093/g3journal/jkab211

**Published:** 2021-06-18

**Authors:** Sara J Hanson, Eoin Ó Cinnéide, Letal I Salzberg, Kenneth H Wolfe, Jamie McGowan, David A Fitzpatrick, Kate Matlin

**Affiliations:** Department of Molecular Biology, Colorado College, Colorado Springs, CO 80903, USA; School of Medicine, UCD Conway Institute, University College Dublin, Dublin 4, Ireland; School of Medicine, UCD Conway Institute, University College Dublin, Dublin 4, Ireland; School of Medicine, UCD Conway Institute, University College Dublin, Dublin 4, Ireland; Genome Evolution Laboratory, Department of Biology, Maynooth University, Maynooth, Ireland; Kathleen Lonsdale Institute for Human Health Research, Maynooth University, Maynooth, Ireland; Genome Evolution Laboratory, Department of Biology, Maynooth University, Maynooth, Ireland; Kathleen Lonsdale Institute for Human Health Research, Maynooth University, Maynooth, Ireland; Department of Molecular Biology, Colorado College, Colorado Springs, CO 80903, USA

**Keywords:** *Ogataea*, population genomics, mating-type switching, interspecies hybridization, chromosomal rearrangements

## Abstract

The methylotrophic yeast *Ogataea polymorpha* has long been a useful system for recombinant protein production, as well as a model system for methanol metabolism, peroxisome biogenesis, thermotolerance, and nitrate assimilation. It has more recently become an important model for the evolution of mating-type switching. Here, we present a population genomics analysis of 47 isolates within the *O. polymorpha* species complex, including representatives of the species *O. polymorpha*, *Ogataea parapolymorpha*, *Ogataea haglerorum*, and *Ogataea angusta*. We found low levels of nucleotide sequence diversity within the *O. polymorpha* species complex and identified chromosomal rearrangements both within and between species. In addition, we found that one isolate is an interspecies hybrid between *O. polymorpha* and *O. parapolymorpha* and present evidence for loss of heterozygosity following hybridization.

## Introduction

The yeast *Ogataea polymorpha* is one of a small number of yeasts in the Pichiaceae family (Shen *et al.* 2016, 2018) with the ability to metabolize methanol as a sole carbon source (Yamada *et al.* 1994; Kurtzman and Robnett 2010). The methylotrophic characteristics of *O. polymorpha* have made it an important model system for examining metabolic processes and peroxisome biology (Siverio 2002; van der Klei and Veenhuis 2002; Yurimoto *et al.* 2002; Hartner and Glieder 2006). In addition, the strongly inducible promoters for genes involved in methanol metabolism have made it a useful tool for recombinant protein production (Gellissen and Melber 1996).

*O. polymorpha* has also emerged as a model for the evolution of yeast mating-type switching. Yeast mating occurs between haploid cells of the opposite mating type (a and α), which is designated by the transcription factors present at the mating-type locus (*MATa* or *MATα*). When a haploid cell does not have an available mating partner, it can undergo a programmed DNA rearrangement to displace the genes found at the *MAT* locus and replace them with genes for the opposite mating type. This switching mechanism occurs through a two-locus “flip/flop” system in *O. polymorpha*, in which the *MATa* and *MATα* genes are found separated by 19 kb of sequence and flanked by 2 kb inverted repeat sequences (Hanson *et al.* 2014; Maekawa and Kaneko 2014). The *MAT* region is adjacent to a centromere, resulting in transcriptional silencing of the *MAT* genes closest to the centromere and the designation of mating type by the distal *MAT* genes. Mating-type switching occurs through recombination between the flanking inverted repeats, which causes an inversion of the *MAT* region and a change in mating type. This mode of mating-type switching has been demonstrated in five yeast species (Hanson *et al.* 2014; Maekawa and Kaneko 2014; Riley *et al.* 2016; Yoko-O *et al.* 2019; Wongwisansri *et al.* 2020) and has been inferred by genome structure in 26 additional species, and appears to have evolved independently 11 times (Riley *et al.* 2016; Krassowski *et al.* 2019). Investigation into the genetic diversity in *O. polymorpha* will be a valuable resource for future investigation into its cellular processes and the impacts of mating-type switching on the evolution of its genome.

Exploration of genetic diversity in yeast populations has revealed insights into intraspecific variation and the influence of recombination and selection on genome evolution (Peter and Schacherer 2016). Extensive datasets have been created to examine population dynamics in *Saccharomyces cerevisiae* (Strope *et al.* 2015; Gallone *et al.* 2016; Gonçalves *et al.* 2016; Zhu *et al.* 2016; Peter *et al.* 2018), *Schizosaccharomyces pombe* (Fawcett *et al.* 2014; Jeffares *et al.* 2015), and pathogenic yeasts (Ford *et al.* 2015; Hirakawa *et al.* 2015; Carreté *et al.* 2018; Ropars *et al.* 2018; Wang *et al.* 2018; Chow *et al.* 2020). Population genomics have also been performed on a variety of nonmodel yeast species (Almeida *et al.* 2014; Bergström *et al.* 2014; Friedrich *et al.* 2015; Ortiz-Merino *et al.* 2018), including the methylotroph *Komagataella phaffii* (Braun-Galleani *et al.* 2019) and nonmethylotrophic yeasts in the Pichiaceae family (Douglass *et al.* 2018; Gounot *et al.* 2020).

In this study, we sequenced 47 isolates of yeast in the *O. polymorpha* species complex, representing four species (*O. polymorpha*, *Ogataea parapolymorpha*, *Ogataea angusta*, and *Ogataea haglerorum*). We examined the genome-wide genetic diversity across the isolates, as well as the genetic diversity in functional regions including centromeres, telomeres, and the *MAT* region. We further identified evidence of chromosomal rearrangements within and between species and found that one isolate is a diploid interspecies hybrid between *O. polymorpha* and *O. parapolymorpha*.

## Materials and methods

### Genomic DNA extractions

Overnight cultures of yeast were grown in YPD broth (1% w/v yeast extract, 2% w/v peptone, 2% w/v glucose) in a 37°C shaking incubator. Genomic DNA was extracted from the yeast samples using an Epicentre MasterPure Yeast DNA Purification Kit (Lucigen) according to manufacturer’s instructions or by acid-washed bead homogenization, phenol chloroform extraction, and concentration using a Genomic DNA Clean & Concentrator-10 kit (Zymo Research).

For MinION library preparation, CBS1977 genomic DNA was extracted using the Qiagen Genomic Tip 100/G kit according to manufacturer’s instructions with the following modifications: a final wash step of 2 × 1 ml ethanol, during which the DNA pellet was transferred to an Eppendorf tube; the sample was then vacuum-centrifuge dried.

### Genome sequencing and assembly

Genomic DNA libraries were prepared and sequenced by BGI Tech Solutions (Hong Kong). Approximately 100X genome coverage with 150-bp paired-end reads were generated for each isolate using an Illumina HiSeq 4000. Reads were assembled using SPAdes version 3.11 (Bankevich *et al.* 2012) and contaminating sequences were removed using coverage-versus-length plots (Douglass *et al.* 2019). Assembly statistics were generated using QUAST version 5.0.2 (Gurevich *et al.* 2013). Structural variation was assessed by generating genome-wide pairwise dot plots using D-Genies version 1.2.0 (Cabanettes and Klopp 2018).

For MinION sequencing library preparation, 400 ng of CBS1977 DNA was barcoded using a Rapid Barcoding Kit (SQK-RBK004). The final sample was concentrated using AMPure XP beads and re-eluted in 10 mM Tris 50 mM NaCl. The library sample was applied to a MinION flow cell (version FLO-MIN106) and run for 50 hours. Approximately 332,000 reads were generated, read quality was assessed using NanoPlot version 1.30.1 and all reads <1000 bp were filtered using NanoFilt version 2.7.1. The genome of CBS1977 was assembled using Canu version 1.8 (Koren *et al.* 2017), using the following command: “canu -p canu -d canu_run2_fitlered_reads genomeSize = 8.9m corOutCoverage = 200 ‘batOptions=-dg 3 -db 3 -dr 1 -ca 500 -cp 50’ -nanopore-raw CBS1977_all_filtered_q7.fastq &.” Truncated or frameshifted protein-coding ORFs were predicted using IDEEL (Watson 2018). The assembly was polished two times with Pilon version 1.23 (Walker *et al.* 2014) using Illumina sequencing data from CBS1977 (this study). IDEEL plots were generated to evaluate the expected ORF length.

### Genome annotation

Gene annotation on each assembled genome was performed using Augustus version 3.3.3 (Stanke and Morgenstern 2005) with the following parameters: –strand=both –species=lodderomyces_elongisporus. tRNAs were annotated using tRNA-scanSE version 2.0.5 (Chan and Lowe 2019) with -E parameter. *MAT* regions for each genome were identified by performing a local blastn version 2.2.31 (Altschul *et al.* 1990) search using the *O. polymorpha* NCYC495 *MAT* region sequence as a query. Identified *MAT* region annotations were manually curated using Artemis version 18.0.3 (Carver *et al.* 2012).

### Variant calling

Variant calling was performed within each species using the previously published *O. polymorpha* NCYC495 (Riley *et al.* 2016) or *O. parapolymorpha* DL-1 (Ravin *et al.* 2013) genome assemblies, and the *O. angusta* 61-244 (Oang9) or *O. haglerorum* 81-453-3 (Ohag10) genome assemblies from this study. Reference genome FASTA files were indexed with BWA version 0.7.17 (Li and Durbin 2009), SAMtools version 1.10 (Li *et al.* 2009), and Picard version 2.22.5 (http://broadinstitute.github.io/picard/). Sequencing reads were mapped to the reference fasta files using the BWA-MEM algorithm with -M, -Y, and -R parameters. BAM alignment files generated by bwa were converted to SAM format, sorted, and indexed using SAMtools. Deduplication and indexing was performed using Picard. Structural variants were identified with Delly version 0.8.3 (Rausch *et al.* 2012), filtered for “PASS,” and assessed through the manual confirmation of evidence in read mapping.

Variants were called using GATK version 4 (Poplin *et al.* 2017) HaplotypeCaller and compiled across isolates for each species using CombineGVCFs. VCF files for each isolate were generated from the GVCF files using GenotypeGVCF. Heterozygous variants were filtered from the dataset using VariantFiltration and SelectVariants. SNP density, nucleotide diversity (P), and Tajima’s D were calculated using VCFtools version 0.1.16 (Danecek *et al.* 2011).

### Population structure analysis

VCF files were converted to PHYLIP SNP alignments using a python script (Ortiz 2019). Maximum likelihood phylogenetic analysis was performed using PhyML version 3.1 (Guindon and Gascuel 2003) with GTR substitution model (Waddell and Steel 1997) and 100 bootstrap replicates.

### Phylogenomics

A phylogenomics analysis was performed on all *O. polymorpha* species complex isolates, the previously published *O. polymorpha* NCYC495 genome (Riley *et al.* 2016), an additional 20 *Ogataea* species (Shen *et al.* 2018), and *Pichia kudriavzevii* (Douglass *et al.* 2018) as an outgroup. A second analysis was also performed on a dataset containing one representative isolate for each of the newly sequenced *O. angusta* (Oang9), *O. haglerorum* (Ohag10), and *O. parapolymorpha* (Opar4) species. BUSCO analysis (Waterhouse *et al.* 2018) revealed 1148 BUSCO families present in all isolates and 1278 BUSCO families that are present in all 25 species. Each BUSCO family was individually aligned with MUSCLE (Edgar 2004) and trimmed using trimAl (Capella-Gutierrez *et al.* 2009) with the parameter “-automated1” to remove poorly aligned regions. Trimmed alignments were concatenated together resulting in a supermatrix alignment of 632,568 amino acids for the analysis including all isolates, and 644,187 amino acids for the analysis with one representative per species. To speed up computation, phylogenetically uninformative sites were removed from the alignment that contained one representative per species generating a final alignment of 319,116 amino acids. Maximum-likelihood (ML) phylogenetic reconstruction was performed using IQ-TREE (Nguyen *et al.* 2015) with the LG+F+R4 model, which was the best-fit model according to ModelFinder (Kalyaanamoorthy *et al.* 2017), and 100 bootstrap replicates were undertaken to infer branch support values. For the alignment that contained all isolates, an approximately maximum-likelihood phylogeny, and local support values were generated using FastTree (Price *et al.* 2010). Both phylogenies were visualized and annotated using the Interactive Tree of Life (iTOL) (Letunic and Bork 2019).

### Hybrid genome analysis

The size of the genome assembly for CBS1977 indicated that it was likely a diploid. BLAST analysis of segments of the genome assembly suggested that it resulted from a hybridization event between *O. polymorpha* and *O. parapolymorpha.* To determine parental contributions to the diploid hybrid CBS1977 genome, SWeBLAST (Fourment *et al.* 2008) was used to perform nucleotide BLAST on 1000 bp windows of each MinION and Illumina contig in the genome assembly against the *O. polymorpha* NCYC495 and *O. parapolymorpha* DL-1 reference genomes with a 97% nucleotide identity cutoff.

### Data availability

The genome sequence data, genome assemblies, and annotations generated in this study were submitted to the NCBI database under the BioProject accession number PRJNA706707. BioSample accessions are SAMN18128820-SAMN18128866, SRA accessions are SRR13943463-SRR13943509 and SRR13944969, and genome assembly and annotation accessions are JAHKSL000000000, JAHLUA000000000-JAHLUZ000000000, and JAHLVA000000000-JAHLVT000000000).

Supplementary material is available at *G3* online.

## Results and discussion

### Genome sequencing and assembly of 47 *Ogataea* isolates

We obtained 47 yeast isolates identified as *O. polymorpha* in the Phaff collection (University of California-Davis, CA, USA), the CBS collection (Westerdijk Fungal Diversity Institute, Utrecht, The Netherlands), and the NRRL collection (Agricultural Research Service, National Center for Agricultural Utilization Research, Peoria, IL, USA) (Table 1). Many of the isolates were originally isolated from decaying plant matter, soil, and insect frass, consistent with their methylotrophic characteristics, as the methanol and methoxy groups in decaying plant matter can be metabolized as a source of carbon (Fall and Benson 1996; Kurtzman and Robnett 2010). In addition, several clinical and agricultural samples were included from an infected human knee, catheter fungemia, swine intestine, and cow mastitis. The geographic distribution of the isolates included North America, Europe, Australia, and South Africa.

**Table 1:** *Ogataea* isolates sequenced in study

Strain	Species	Strain ID	Source^*c*^	Location^*c*^	Ploidy
Opol1*^a^*	*O. polymorpha*	CBS4732/Y-5445/ATCC 34438	Soil	Brazil	Haploid
Opol2	*O. polymorpha*	CBS1976/NRRL Y-1798/ ATCC 14754/NCYC495	Spoiled Florida orange juice	USA	Haploid
Opol3	*O. polymorpha*	Phaff 72-225	Glutinous/nonglutinous rice	USA	Haploid
Opol4	*O. polymorpha*	NRRL Y-2423	Swine intestinal tract	Portugal	Haploid
Opol5	*O. polymorpha*	CBS8852/NRRL Y-27293.	Knee replacement	Worcester, MA, USA	Haploid
Opol6	*O. polymorpha*	NRRL Y-27863/ATCC MYAA-3665	Patient's blood, catheter infection	Chicago, IL, USA	Haploid
Opol7	*O. polymorpha*	NRRL Y-6005	Waste liquid from olive processing	Spain	Haploid
Opol8	*O. polymorpha*	NRRL YB-179	Soil	Costa Rica	Haploid
Opol9	*O. polymorpha*	CBS5032	Maize meal	South Africa	Haploid
Opol10	*O. polymorpha*	CBS7031	Soil	Unknown	Haploid
Opol11	*O. polymorpha*	CBS7239	Catalase-negative mutant of CBS4732 (PMID 7000025)	Germany	Haploid
Opar1*^a^*	*O. parapolymorpha*	CBS12304/NRRL YB-1982	Insect frass, quaking aspen	Duluth, MN, USA	Haploid
Opar2	*O. parapolymorpha*	Phaff 73-26	Soil	MA, USA	Haploid
Opar3 × Opol	Hybrid (*O. polymorpha* × *O. parapolymorpha*)	CBS1977	Milk from cow with mastitis	UK	Diploid
Opar4	*O. parapolymorpha*	CBS11895/NRRL Y-7560/ ATCC 26012	Soil	Cambridge, MA, USA	Haploid
Oang1	*O. angusta*	Phaff 50-165/NRRL Y-2217	*Drosophila pseudoobscura*	Jacksonville, CA, USA	Haploid
Oang2	*O. angusta*	Phaff 50-97/NRRL Y-2212	*D. pseudoobscura*	Keen Camp, CA, USA	Haploid
Oang3	*O. angusta*	Phaff 51-138	*D. pseudoobscura*	Mather, CA, USA	Haploid
Oang4	*O. angusta*	Phaff 51-177	*Aulacigaster* sp.	Mather, CA, USA	Haploid
Oang5	*O. angusta*	Phaff 52-251	*D. pseudoobscura*	Mather, CA, USA	Haploid
Oang6	*O. angusta*	Phaff 60-394/ATCC 24190	*D. pseudoobscura*	Winters, CA, USA	Haploid
Oang7	*O. angusta*	Phaff 61-224	*Aulacigaster* sp.	Gualala River, CA, USA	Haploid
Oang8	*O. angusta*	Phaff 61-235	*Drosophila viridis*	Gualala River, CA, USA	Haploid
Oang9*^b^*	*O. angusta*	Phaff 61-244	*Aulacigaster* sp.	Gualala River, CA, USA	Haploid
Oang10	*O. angusta*	CBS2575/NCYC1450	*Aulacigaster* sp.	USA	Haploid
Ohag1	*O. haglerorum*	Phaff 78-557.3	*Opuntia stricta*	Hemmant, Queensland, AU	Haploid
Ohag2	*O. haglerorum*	Phaff 79-204.41	*O. stricta*	Hemmant, Queensland, AU	Haploid
Ohag3	*O. haglerorum*	Phaff 81-408.1	*O. phaeacantha*	Saguaro Natl. Monument West, AZ, USA	Haploid
Ohag4	*O. haglerorum*	Phaff 81-410	*O. phaeacantha*	Saguaro Natl. Monument West, AZ, USA	Haploid
Ohag5	*O. haglerorum*	Phaff 81-419.3	*O. phaeacantha*	Bear Canyon, Tucson, AZ, USA	Haploid
Ohag6	*O. haglerorum*	Phaff 81-419.5	*O. phaeacantha*	Bear Canyon, Tucson, AZ, USA	Haploid
Ohag7	*O. haglerorum*	Phaff 81-433.4	*O. phaeacantha*	Santa Rita Mountains, Tucson, AZ, USA	Haploid
Ohag8	*O. haglerorum*	Phaff 81-436.3	*O. phaeacantha*	Santa Rita Mountains, Tucson, AZ, USA	Haploid
Ohag9	*O. haglerorum*	Phaff 81-440.2	*O. phaeacantha*	Santa Rita Mountains, Tucson, AZ, USA	Haploid
Ohag10*^b^*	*O. haglerorum*	Phaff 81-453.3	*O. phaeacantha*	Near Sells, AZ, USA	Haploid
Ohag11	*O. haglerorum*	Phaff 81-461.3	*O. phaeacantha*	Near Sells, AZ, USA	Haploid
Ohag12	*O. haglerorum*	Phaff 81-463.1	*O. phaeacantha*	Near Sells, AZ, USA	Haploid
Ohag13	*O. haglerorum*	Phaff 81-471.3	*O. phaeacantha*	Rincon Mountains, AZ, USA	Haploid
Ohag14	*O. haglerorum*	Phaff 81-480	*O. phaeacantha*	Rincon Mountains, AZ, USA	Haploid
Ohag15	*O. haglerorum*	Phaff 83-405.1	*O. phaeacantha*	Tucson Mountains, AZ, USA	Haploid
Ohag16	*O. haglerorum*	Phaff 83-425.4	*O. phaeacantha*	Tucson, AZ, USA	Haploid
Ohag17	*O. haglerorum*	Phaff 83-437.2.1	*O. phaeacantha*	Santa Rita Mountains, Tucson, AZ, USA	Haploid
Ohag18	*O. haglerorum*	Phaff 83-437.2.2	*O. phaeacantha*	Santa Rita Mountains, Tucson, AZ, USA	Haploid
Ohag19	*O. haglerorum*	Phaff 83-442.1	*O. phaeacantha*	AZ, USA	Haploid
Ohag20	*O. haglerorum*	Phaff 83-471.3	*O. phaeacantha*	Santa Catalina Mountains, AZ, USA	Haploid
Ohag21	*O. haglerorum*	Phaff 83-474.2	*O. phaeacantha*	Pima Canyon, Tucson, AZ, USA	Haploid
Ohag22	*O. haglerorum*	Phaff 83-476.5	*O. phaeacantha*	Pima Canyon, Tucson, AZ, USA	Haploid

a Type strain.

b Reference strain for varaint analysis.

c Information provided in culture collection database.

Following short-read sequencing and genome assembly of the isolates, we identified them as representatives of four distinct species previously described as members of the *O. polymorpha* species complex (Suh and Zhou 2010; Kurtzman 2011; Naumov *et al.* 2017): *O. polymorpha* (11 isolates), *O. angusta* (10 isolates), *O. haglerorum* (22 isolates), and *O. parapolymorpha* (3 isolates) (Supplementary Figure S1). Post-zygotic isolation has been described between *O. polymorpha*, *O. angusta*, and *O. haglerorum*, for which hybrids show reduced spore viability (Naumov *et al.* 1997). *O. parapolymorpha* industrial strain DL-1 is “semi-sterile” (Lahtchev *et al.* 2002) due to a mutation in the nitrogen-sensing transcription factor *EFG1*, although it is able to form rare diploids when crossed with *O. polymorpha* (Hanson *et al.* 2017). Of the 3 *O. parapolymorpha* isolates, 2 showed very high nucleotide similarity (>99%) to DL-1. The third isolate, Opar1 (CBS12304^T^), has an intact *EFG1* locus without the single nucleotide insertion found in DL-1. This may account for the previously described homothallic behavior of this isolate (Suh and Zhou 2010; Kurtzman 2011).

The genome assemblies for all four species were similar in their overall size (8.8–8.9 Mb; Table 2). The N50 of the genome assemblies for the haploid strains ranged from 263.7 to 856.3 kb and the number of contigs ranged from 45 to 154 (Table 2). The GC content of *O. parapolymorpha* strain DL-1 (47.8%) has previously been described as higher than many other yeast species such as *S. cerevisiae* (38%; Peter *et al.* 2018), which may be related to its thermotolerant characteristics (Ravin *et al.* 2013). We found that the GC content for the *O. polymorpha* isolates was similarly high (47.7–47.8%), and that the GC content for the *O. angusta* and *O. haglerorum* isolates was even higher at 49.4–49.5% (Table 2).

**Table 2:** *Ogataea* genome assembly and annotation statistics

Strain	Strain ID	Genome length (Mb)	N50 (kb)	# Contigs	% GC	tRNA Genes	Protein-coding genes
Opol1*^a^*	CBS4732/Y-5445/ATCC 34438	8.93	556.0	67	47.7	97	5,442
Opol2	CBS1976/NRRL Y-1798/ATCC 14754/NCYC495	8.93	556.4	64	47.7	96	5,446
Opol3	Phaff 72-225	8.95	788.8	45	47.7	97	5,444
Opol4	NRRL Y-2423	8.92	608.6	79	47.7	98	5,436
Opol5	CBS8852/NRRL Y-27293.	8.95	636.5	64	47.7	99	5,451
Opol6	NRRL Y-27863/ATCC MYAA-3665	8.97	616.1	51	47.7	97	5,454
Opol7	NRRL Y-6005	8.91	631.1	51	47.7	96	5,431
Opol8	NRRL YB-179	8.93	552.0	85	47.7	99	5,440
Opol9	CBS5032	8.9	626.9	52	47.8	99	5,417
Opol10	CBS7031	8.97	636.4	79	47.7	99	5,455
Opol11	CBS7239	8.94	516.4	67	47.7	97	5,442
Opar1*^a^*	CBS12304/NRRL YB-1982	8.87	557.1	55	47.7	97	5,417
Opar2	Phaff 73-26	8.92	263.7	112	47.8	99	5,456
Opar3 × Opol	CBS1977	14.88	30.1	1521	47.9	155	9,920
Opar4	CBS11895/NRRL Y-7560/ATCC 26012	8.92	618.1	87	47.8	97	5,424
Oang1	Phaff 50-165/NRRL Y-2217	8.88	848.7	54	49.5	97	5,409
Oang2	Phaff 50-97/NRRL Y-2212	8.88	655.9	73	49.4	97	5,437
Oang3	Phaff 51-138	8.89	553.9	127	49.5	100	5,430
Oang4	Phaff 51-177	8.89	743.6	96	49.5	97	5,443
Oang5	Phaff 52-251	8.89	651.6	108	49.5	97	5,437
Oang6	Phaff 60-394/ATCC 24190	8.88	856.3	57	49.5	97	5,419
Oang7	Phaff 61-224	8.91	651.8	154	49.4	99	5,453
Oang8	Phaff 61-235	8.9	557.2	107	49.5	97	5,452
Oang9*^b^*	Phaff 61-244	8.91	850.2	101	49.4	97	5,446
Oang10	CBS2575/NCYC1450	8.91	787.0	109	49.5	97	5,441
Ohag1	Phaff 78-557.3	8.85	583.5	50	49.4	97	5,390
Ohag2	Phaff 79-204.41	8.85	555.7	49	49.4	98	5,392
Ohag3	Phaff 81-408.1	8.87	556.8	68	49.4	99	5,393
Ohag4	Phaff 81-410	8.86	416.0	73	49.4	97	5,412
Ohag5	Phaff 81-419.3	8.87	465.9	109	49.4	103	5,419
Ohag6	Phaff 81-419.5	8.86	632.4	63	49.4	100	5,415
Ohag7	Phaff 81-433.4	8.86	583.9	65	49.4	100	5,401
Ohag8	Phaff 81-436.3	8.87	556.4	71	49.4	97	5,407
Ohag9	Phaff 81-440.2	8.87	555.9	75	49.4	97	5,404
Ohag10*^b^*	Phaff 81-453.3	8.88	556.7	73	49.4	99	5,411
Ohag11	Phaff 81-461.3	8.85	579.9	52	49.4	99	5,395
Ohag12	Phaff 81-463.1	8.86	584.1	62	49.4	97	5,404
Ohag13	Phaff 81-471.3	8.86	556.9	64	49.4	97	5,413
Ohag14	Phaff 81-480	8.86	437.7	66	49.4	97	5,423
Ohag15	Phaff 83-405.1	8.86	466.2	66	49.4	97	5,408
Ohag16	Phaff 83-425.4	8.87	583.5	74	49.4	99	5,422
Ohag17	Phaff 83-437.2.1	8.86	466.2	56	49.4	98	5,413
Ohag18	Phaff 83-437.2.2	8.86	497.3	57	49.4	98	5,401
Ohag19	Phaff 83-442.1	8.86	584.3	59	49.4	97	5,402
Ohag20	Phaff 83-471.3	8.86	556.4	65	49.4	97	5,412
Ohag21	Phaff 83-474.2	8.86	582.9	71	49.4	99	5,402
Ohag22	Phaff 83-476.5	8.89	632.2	89	49.4	101	5,406

a Type strain.

b Reference strain for varaint analysis.

### Phylogenomic analysis resolves interspecies relationships in the *O. polymorpha* species complex

To establish the relationships among the four species in the *O. polymorpha* species complex, and their relationship to other species in the *Ogataea* genus, we performed a phylogenomic analysis using shared Benchmarking Universal Single-Copy Orthologs (BUSCO) (Waterhouse *et al.* 2018). The annotation was performed using the 2137 genes in the Saccharomycetes BUSCO set and was highly complete (96.7–97.3%) for each of the 47 genomes sequenced (Supplementary Figure S2).

**Figure 1:**
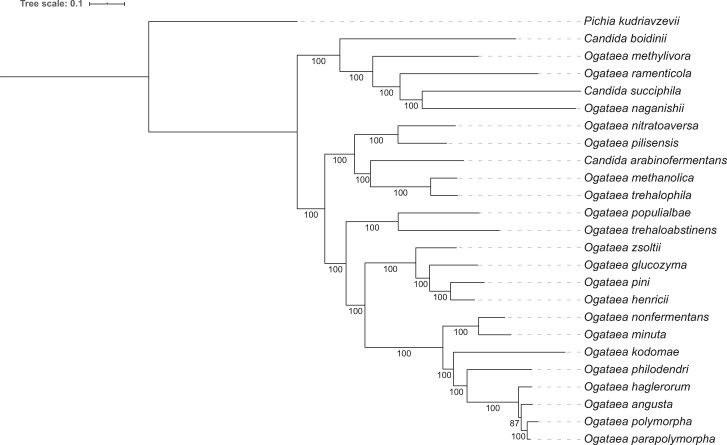
Relationship of the *O. polymorpha* species complex to other *Ogataea* species. Supermatrix phylogeny of 24 *Ogataea* species derived from 1278 BUSCO families giving an alignment 319,116 amino acids in length. *P. kudriavzevii* is included as an outgroup. Maximum Likelihood phylogeny was reconstructed with IQ-TREE implementing the JTT+F+R5 model. Bootstrap support values are indicated at all nodes.

Maximum likelihood analysis of a concatenated alignment of the 1278 BUSCO sequences shared across 25 yeast species resolved the relationship among the four members of the *O. polymorpha* species complex (Figure 1), which matches the relationships shown by previous analysis of the rDNA and translation elongation factor-1α sequences (Naumov *et al.* 2017). The topology of the rest of the tree is consistent with previous analysis (Shen *et al.* 2018).

### Population structure in the *O. polymorpha* species complex

Previous studies showed that species within the *O. polymorpha* species complex occupy different environmental niches, with specificity of *O. parapolymorpha* to insect-damaged trees (Kurtzman 2011), *O. haglerorum* to rotting *Opuntia* cacti (Naumov *et al.* 2017), and *O. angusta* to insects (Suh and Zhou 2010). In contrast, *O. polymorpha* has been described as a generalist, having been isolated from a variety of sources and with broad geography (Kurtzman 2011). The information available for our sequenced isolates are consistent with these observations, as the *O. polymorpha* isolates were sampled from clinical, soil, and agricultural sources from a broad geographic distribution, the *O. haglerorum* isolates were sampled from rotting *Opuntia* samples in Australia and Arizona, and *O. angusta* isolates were sampled from *Drosophila* and *Aulacigaster* insect species (Table 1).

For each of the *Ogataea* species, we examined genomic diversity by aligning reads to a reference genome [previously published NCYC495 for *O. polymorpha* (Riley *et al.* 2016) and DL-1 for *O. parapolymorpha* (Ravin *et al.* 2013) and Oang9 for *O. angusta* and Ohag10 for *O. haglerorum* generated in this study]. Single nucleotide polymorphisms (SNPs) and insertion-deletion (indels) from the mapped reads were quantified (Table 3). We then analyzed the population structure for three of the four *Ogataea* species in our study using maximum likelihood analysis of SNP alignments (*O. parapolymorpha* was excluded due to the low number of representative isolates).

**Table 3:** Summary of genetic variation in *Ogataea*

	Total		Per kb	
Strain	SNP	Indel	SNP	Indel
	**Relative to NCYC495**			
Opol1*^a^*	26,824	1,275	3.00	0.14
Opol2	9,609	661	1.08	0.07
Opol3	42,399	1,820	4.74	0.20
Opol4	31,882	1,425	3.57	0.16
Opol5	35,033	1,578	3.91	0.18
Opol6	38,878	1,665	4.34	0.19
Opol7	42,586	1,881	4.78	0.21
Opol8	36,772	1,707	4.12	0.19
Opol9	33,877	1,546	3.80	0.17
Opol10	49,307	2,049	5.50	0.23
Opol11	27,073	1,219	3.03	0.14
	**Relative to DL-1**			
Opar1*^a^*	113,030	3,221	12.74	0.36
Opar2	207	569	0.02	0.06
Opar4	197	558	0.02	0.06
	**Relative to Oang9**			
Oang1	52,192	1,991	5.88	0.22
Oang2	47,937	1,868	5.40	0.21
Oang3	48,832	1,789	5.49	0.20
Oang4	48,960	1,788	5.51	0.20
Oang5	48,844	1,795	5.49	0.20
Oang6	47,059	1,742	5.30	0.20
Oang7	48,613	1,778	5.46	0.20
Oang8	47,735	1,642	5.36	0.18
Oang9*^b^*	n/a	n/a	n/a	n/a
Oang10	49,183	1,766	5.52	0.20
	**Relative to Ohag10**			
Ohag1	19,971	979	2.26	0.11
Ohag2	19,938	931	2.25	0.11
Ohag3	20,581	1,055	2.32	0.12
Ohag4	20,508	974	2.31	0.11
Ohag5	20,238	1,009	2.28	0.11
Ohag6	20,189	1,001	2.28	0.11
Ohag7	19,665	989	2.22	0.11
Ohag8	20,899	1,026	2.36	0.12
Ohag9	20,725	1,059	2.34	0.12
Ohag10*^b^*	n/a	n/a	n/a	n/a
Ohag11	15,424	821	1.74	0.09
Ohag12	19,523	974	2.20	0.11
Ohag13	20,997	1,053	2.37	0.12
Ohag14	20,793	1,003	2.35	0.11
Ohag15	20,447	1,002	2.31	0.11
Ohag16	20,234	1,015	2.28	0.11
Ohag17	19,953	943	2.25	0.11
Ohag18	19,913	934	2.25	0.11
Ohag19	20,699	1,043	2.34	0.12
Ohag20	20,788	1,078	2.35	0.12
Ohag21	20,663	1,047	2.33	0.12
Ohag22	19,534	969	2.21	0.11

a Type strain.

b Reference strain for varaint analysis.

**Figure 2:**
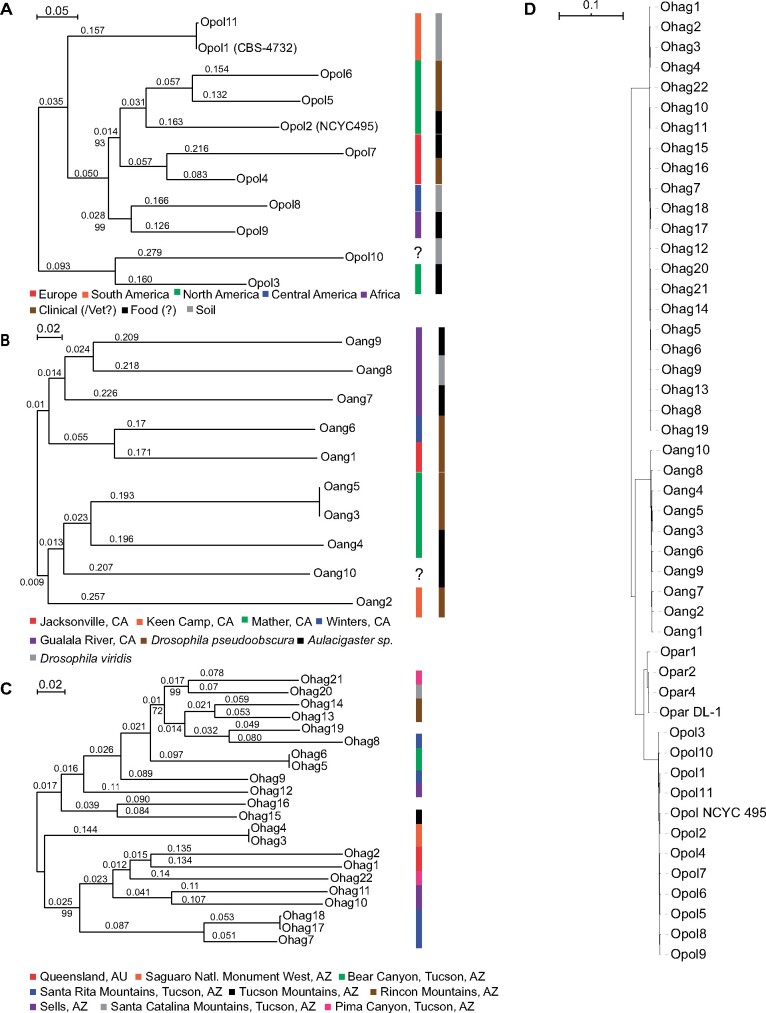
Population structure of the *O. polymorpha* species complex. Maximum likelihood phylogenies created using SNP alignments for (A) *O. polymorpha*, (B) *O. angusta*, and (C) *O. haglerorum* isolates. Bootstrap support was 100% except where indicated below the branch, and branch lengths are given above each branch. Geographic information for isolates is indicated using colored boxes. (D) Supermatrix phylogeny of 48 *Ogataea* isolates generated using 1,148 BUSCO families.

The population structure of *O. polymorpha* corresponded better to geography than the source from which the samples were isolated (Figure 2A). North American samples grouped together, except for Opol3, for which the isolation location is unclear in the Phaff collection database. The clustering of isolates was also consistent with geography for two European (Opol4 and Opol7) and two South American isolates, although Opol11 is a derivative of Opol1 (CBS4732). Two clinical samples isolated from the United States (Opol5 and Opol6) were grouped together and are most closely related to the industrial strain NCYC495 (Opol2).

*O. angusta* and *O. haglerorum* isolates have a much more limited geographic distribution (Table 1), and did not show a high degree of population structure (Figure 2, B and C). The *O. angusta* isolates were obtained from insect samples in northern California, near Sacramento. The topology of the *O. angusta* tree shows that samples collected from the same location are more similar to one another independent of the species of insect from which they were isolated (Figure 2B). *O. haglerorum* isolates were sampled from *Opuntia* cacti in southern Arizona, except for two from Queensland, Australia. Although the two Australian isolates (Ohag1 and Ohag2) group together, the population structure of the other isolates do not correspond to geography. For example, isolates from the Santa Rita Mountains (Ohag7, Ohag8, Ohag9, Ohag17, and Ohag18) do not form a monophyletic group (Figure 2C). The Australian samples do not show a high amount of divergence from the rest of the samples, which likely reflects the introduction of *Opuntia* species to Australia from their native United States with the last two to three centuries (Friedel 2020).

### Evidence for structural variation within and between species

**Figure 3:**
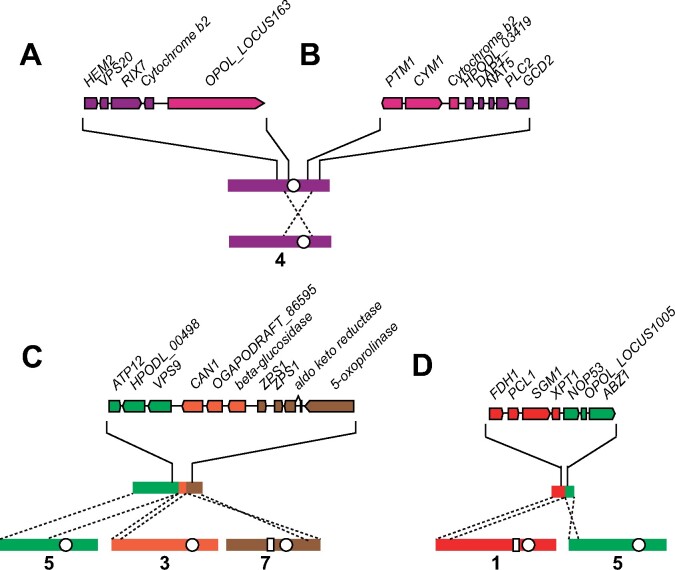
Structural rearrangements in *O. polymorpha*. Chromosomal breakpoints identified in *O. polymorpha* isolate Opol9 (CBS5032) on (A) NODE_10, (B) NODE_5, and (C) NODE_2, and in *O. polymorpha* isolate Opol4 (NRRL Y-2423) on (D) NODE_14 are detailed. Chromosomes are numbered based on *O. polymorpha* NCYC495 genome assembly (shown at the bottom in each panel) and color-coding of genes corresponds to their locations in the NCYC495 genome. White circles indicate the location of centromeres and white boxes indicate the location of a genomic repeat sequence that is found on four chromosomes in the NCYC495 genome.

Structural variation, including inversions, translocations, copy number variations, and fusions/fissions, has roles in adaptation and speciation by impacting gene expression and recombination (Mérot *et al.* 2020). Isolates of *O. polymorpha*, *O. parapolymorpha*, and *O. haglerorum* show an overall high amount of synteny, with very few structural rearrangements. Two isolates of *O. polymorpha* contained rearrangements relative to the NCYC495 reference genome. Opol9 (CBS5032) has a 335-kb pericentromeric inversion in chromosome 4, as well as two translocations that combine parts of chromosomes 3, 5, and 7 (Figure 3, A–C). Opol4 contains a translocation between chromosomes 1 and 5 (Figure 3D). These rearrangements do not involve repetitive genomic elements; most occur in intergenic regions, while the Opol9 chromosome 4 inversion breakpoints occur within the cytochrome b2 locus (*CYB2*) (Figure 3, A and B). The inversion interrupts the 1377 bp *CYB2* locus into a 654 and 723 bp loci. One of the translocation breakpoints occurs adjacent to the *ZPS1* sequence, which is a GPI-anchored protein that responds to low zinc conditions that is present in two tandem copies on chromosome 7 (Figure 3C).

**Figure 4:**
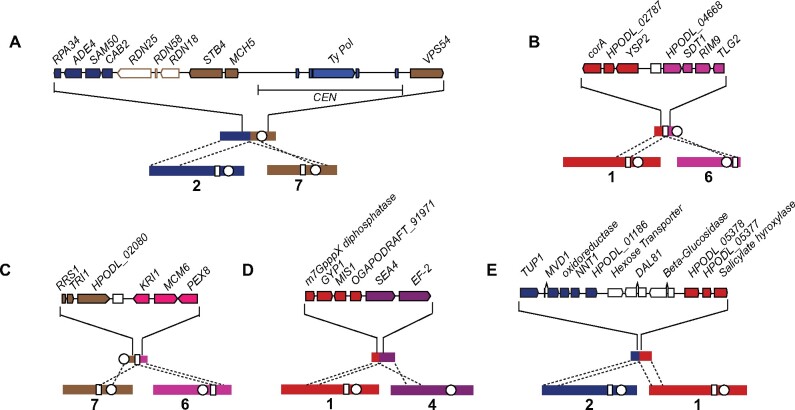
Structural rearrangements in *O. haglerorum*. Chromosomal breakpoints identified in *O. haglerorum* isolates (A) Ohag3 (81-408-1) on NODE_5, (B) Ohag10 (81-453-3) on NODE_15 and Ohag11 (81-461-3) on NODE_16, (C) Ohag21 (83-474-2) on NODE_11, (D) Ohag17 (83-437-2-1) on NODE_14, and (E) Ohag9 (81-440-2) on NODE_10 are detailed. Chromosomes are numbered based on *O. polymorpha* NCYC495 genome assembly and color-coding of genes corresponds to their locations in the NCYC495 genome. White circles indicate the location of centromeres and white boxes indicate the location of a genomic repeat sequence that is found on four chromosomes in the NCYC495 genome.

In *O. haglerorum*, six isolates have structural rearrangements relative to the *O. polymorpha* NCYC495 reference genome assembly (Figure 4). Three of these rearrangements are translocations that occur in repetitive genomic elements. Ohag3 (Phaff 81-408-1) contains a translocation between chromosomes 2 and 7 at the rDNA locus (Figure 4A), which is found adjacent to a centromere containing repetitive Ty-like retrotransposable and long terminal-repeat elements on chromosome 7 in *O. polymorpha*. For a translocation between chromosomes 1 and 6 that is shared between Ohag10 and Ohag11 (Figure 4B), as well as a translocation between chromosomes 6 and 7 in Ohag21 (Figure 4C), the breakpoints occur at a 1-kb repeat sequence. In NCYC495, this sequence occurs on four chromosomes (chromosomes 1, 2, 6, and 7) with 97–98% nucleotide identity between each copy. Based on the topology of the SNP phylogeny (Figure 2C), the shared translocation in Ohag10 and Ohag11 is likely to be the result of a single rearrangement event. Two isolates have translocations in intergenic regions: Ohag17 contains a translocation between chromosomes 1 and 4 (Figure 4D), and Ohag9 has a translocation between chromosomes 1 and 2 (Figure 4E). In Ohag9, the translocated regions of chromosomes 1 and 2 are separated by three genes that are found on chromosomes 2, 3, and 5 in NCYC495.

**Figure 5:**
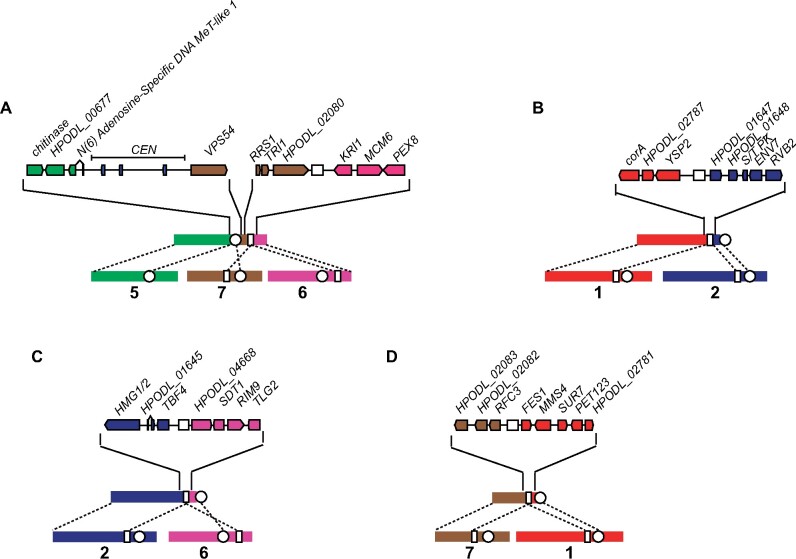
Structural rearrangements in *O. angusta*. Chromosomal breakpoints identified in *O. angusta* isolate Oang4 (51-177) on (A) NODE_1, (B) NODE_5 (C) NODE_2, and (D) NODE_6 are detailed. Chromosomes are numbered based on *O. polymorpha* NCYC495 genome assembly and color-coding of genes corresponds to their locations in the NCYC495 genome. White circles indicate the location of centromeres and white boxes indicate the location of a genomic repeat sequence that is found on four chromosomes in the NCYC495 genome.

In *O. angusta*, multiple translocations are found between the genome of Oang4 (51–177) and the NCYC495 genome (Figure 5). One of the breakpoints is a translocation between the centromeres of chromosomes 5 and 7 (Figure 5B). The rest of the breakpoints are translocations between chromosomes at the same 1-kb repeat region involved in *O. haglerorum* translocations (Figure 5, A–D). Other *O. angusta* isolates have contig breaks in their assemblies at many of the rearrangement breakpoints, leaving the possibility that these rearrangements are shared broadly within the species. These chromosomal rearrangements could explain the reduced spore viability observed in interspecies crosses within the *O. polymorpha* species complex (Naumov *et al.* 1997).

We further examined the genomes of the *O. polymorpha* species complex isolates for evidence of copy number variations (CNVs). We identified two duplications and 22 deletions that were at least 100 bp in length (Supplementary Figure S3). Several of these impact genes with roles in nutrient uptake (*e.g.*, allantoin permease, inositol transporter, amino acid transporter, and sugar transporter). In Oang3, we identified a deletion that included the transcription factor *RME1*, which is required for mating-type switching and mating in *O. polymorpha* (Hanson *et al.* 2017; Yamamoto *et al.* 2017). Loss of this gene potentially impacts the fertility of this isolate. If so, along with the semi-sterility observed in *O. parapolymorpha* DL-1 resulting from the loss of function of the transcription factor *EFG1* (Hanson *et al.* 2017), this would be the second example of fertility loss due to disruption of a transcriptional regulator.

### Genetic variation in the *O. polymorpha* species complex

A concatenated alignment of 1,148 BUSCO amino acid sequences for the *O. polymorpha* species complex isolates indicated a low level of sequence divergence between species (Figure 2D). We used JSpecies (Richter *et al.* 2016) to compare the genome sequences among the 47 isolates and found the pairwise average nucleotide identity between species in the *O. polymorpha* species complex ranges from 86.7% (*O. polymorpha vs O. haglerorum*) to 93.7% (*O. polymorpha vs O. parapolymorpha*).

We assessed the SNP diversity between isolates within each species in the *O. polymorpha* species complex (Table 3). We found that *O. parapolymorpha* isolate Opar1 (CBS12304) showed the highest SNP density (12.74 SNPs/kb) relative to the reference DL-1 genome sequence (Table 3, Supplementary Table S1). *O. haglerorum* isolates demonstrated the least amount of diversity among isolates, with average genome-wide SNP density between 1.74 and 2.36 SNPs/kb relative to Ohag10 (Table 3, Supplementary Table S2). *O. angusta* isolates SNP density ranged between 5.30 and 5.88 SNPs/kb relative to Oang9 (Table 3, Supplementary Table S3), which is higher than what has been described in wild isolates of *S. cerevisiae* (median 4.1 SNPs/kb) (Peter *et al.* 2018). *O. polymorpha* isolate SNP density was 3.00-5.50 SNPs/kb relative to the NCYC495 reference genome sequence (Table 3, Supplementary Table S4), which is comparable to the 1.66-4.66 SNPs/kb observed in the methylotrophic yeast *K. phaffii* (Braun-Galleani *et al.* 2019). The previously published *O. polymorpha* genome sequence (Riley *et al.* 2016), is a laboratory strain derived from CBS1976/NCYC495 (Opol2), and had 1.08 SNPs/kb relative to our Opol2 genome assembly.

**Figure 6:**
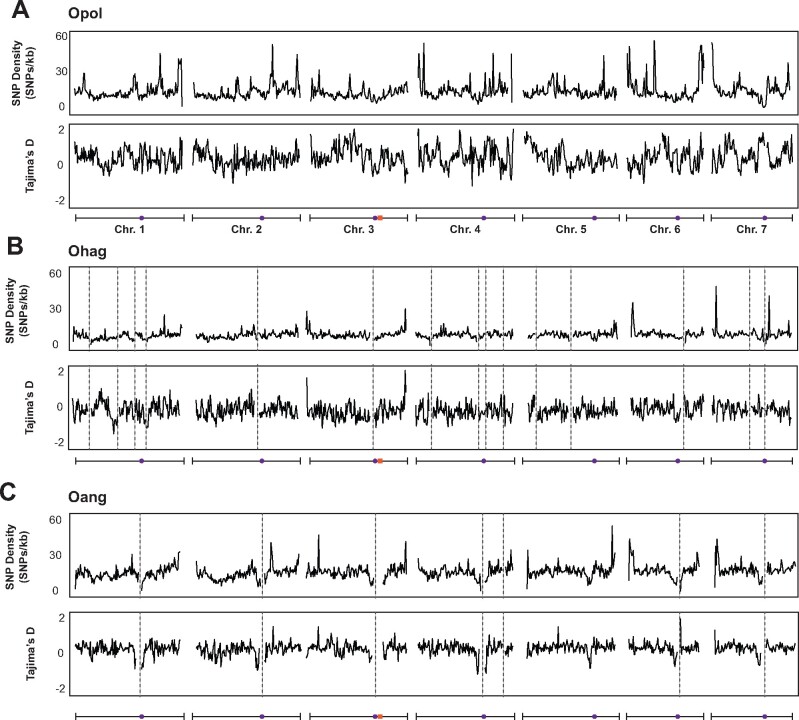
Genome-wide genetic diversity in *Ogataea* species. Plots show density of SNPs (SNPs/kb) and Tajima’s D calculated in 10 kb windows across the genome for all isolates of (A) *O. polymorpha*, (B) *O. haglerorum*, and (C) *O. angusta*. Schematics below each set of plots indicate chromosome with position of centromeres indicated by purple circles and the MAT region indicated by orange boxes. *O. haglerorum* and *O. angusta* contigs greater than 50 kb in length were ordered according to their alignment with the *O. polymorpha* genome, and contig break locations in reference genomes (Oang9 and Ohag10) are indicated by dashed gray lines.

We next examined the distribution of SNPs across the genome for each species (Figure 6). SNP density was similar between chromosomes/contigs for each isolate (Figure 6, Supplementary Tables S1–S4). For each species, SNP density was higher in telomeric regions than the rest of the genome and was higher than or similar to the genome-wide average in centromeric regions (Figures 6 and 8, Supplementary Table S5). We did not observe notable large-scale fluctuations in signatures of selection (Tajima’s D; Figure 6).

### Genetic diversity of the *MAT* region in the *O. polymorpha* species complex

All four species in the *O. polymorpha* species complex have previously been described as homothallic (Suh and Zhou 2010; Kurtzman 2011; Kurtzman *et al.* 2011; Naumov *et al.* 2017), suggesting that they are all able to undergo the flip/flop mating-type switching mechanism previously described in *O. polymorpha* and other species in the *Ogataea* genus (Hanson *et al.* 2014; Maekawa and Kaneko 2014; Krassowski *et al.* 2019; Yoko-O *et al.* 2019; Wongwisansri *et al.* 2020).

**Figure 7:**
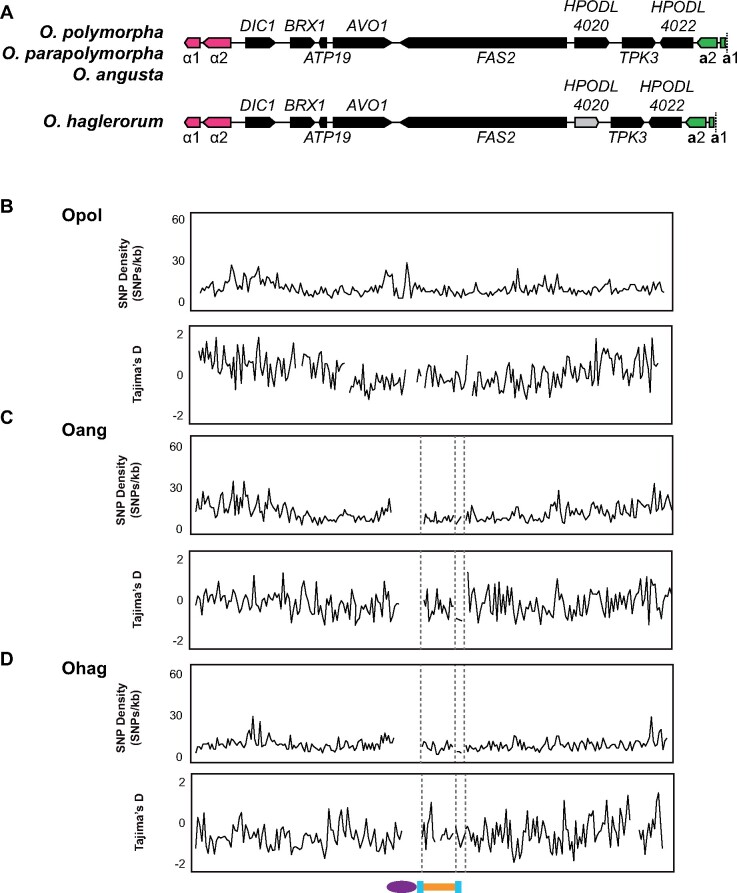
Genetic diversity in the *Ogataea* MAT Region. (A) Schematic of 19 kb MAT region content, drawn to scale. The genes specifying mating-type a are shown in green, and those specifying mating-type a are shown in pink. The gene *HPODL_4020* (shown in gray) is a pseudogene in *O. haglerorum*. Plots show density of SNPs (SNPs/kb) and Tajima’s D calculated in 1 kb windows across the MAT region, and 100 kb upstream and downstream for (B) *O. polymorpha*, (C) *O. angusta*, and (D) *O. haglerorum*. Gray dashed lines indicate contig breaks. Schematic at the bottom shows the location of the centromere (purple), the MAT region (orange), and the inverted repeat sequences (blue).

We annotated the *MAT* region in the newly sequenced *O. angusta* and *O. haglerorum* genomes. The *O. angusta MAT* region was the same size and contained the same set of genes as the previously annotated *O. polymorpha* and *O. parapolymorpha* sequences (Figure 7A) (Hanson *et al.* 2014). The *O. haglerorum MAT* region was ∼500 bp shorter in length (18 *vs* 18.5 kb) due to the *HPODL_4020* locus, which has no known role in sexual processes, undergoing pseudogenization (Figure 7A). The degradation of the *HPODL_4020* sequence was shared across all 22 of the *O. haglerorum* sequences. Other variations in *Ogataea MAT* region gene content have been described. The *O. thermomethanolica MAT* region lacks the *TPK3* locus (Wongwisansri *et al.* 2020), while in *O. minuta* the *MAT* region is longer (23 *vs* 18.5 kb) despite containing the same set of genes. *O. minuta* also has longer inverted repeat sequences that flank the *MAT* region (3.6 *vs* 2 kb) (Yoko-O *et al.* 2019).

Chromosomal inversions are an example of a negative recombination modifier due to the inviability of products that result when meiotic recombination occurs between chromosomes that are heterozygous for the inversion (Schaeffer 2008; Wellenreuther and Bernatchez 2018). The structure of the *MAT* region causes it to be a heterozygous inversion in any diploid cell formed by mating between two cells of opposite mating types. A recombination event in this region in a diploid cell would lead to large-scale chromosomal rearrangements that would result in inviable meiotic products (Hanson *et al.* 2014). We therefore expect that there should be little or no recombination in the region between the *MAT*a and *MAT*α genes in natural populations of *Ogataea.*

**Figure 8:**
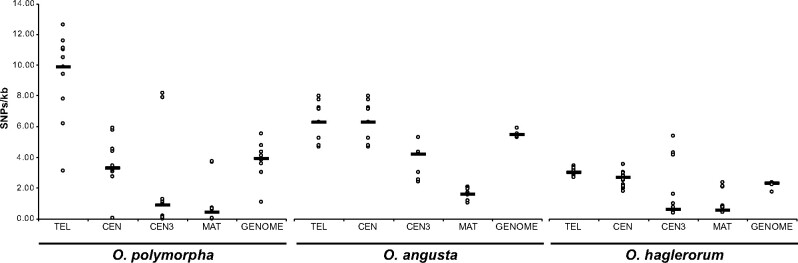
SNP Density at Genomic Features in *Ogataea*. Box and whisker plots show the SNPs/kb at telomeres (within 50 kb of terminal contig ends in genome assemblies), centromeres, at the centromere of chromosome 3, the mating-type locus, and genome-wide for *O. polymorpha*, *O. angusta*, and *O. haglerorum*.

To assess the impact of the *MAT* inversion on genetic diversity, we examined SNP density in the *MAT* region. Overall, SNP density in the *MAT* region (including the adjacent inverted repeat sequence) in each species is lower than in the rest of the genome (Figures 7, B–D and 8, Supplementary Table S5). In addition, for the majority of isolates the centromere adjacent to the *MAT* region (*CEN3*) showed SNP density similar to the *MAT* region (Figures 7, B–D and 8, Supplementary Table S5), much lower than the genome-wide average and the average across all centromeres (Figure 8, Supplementary Table S5).

The pattern of reduced SNP density at the *MAT* region may be due to its centromere proximity. In *S. cerevisiae*, meiotic recombination is suppressed within 10 kb of centromeres (Mancera *et al.* 2008; Pan *et al.* 2011) due to the suppression of Spo11-mediated double-strand breaks by the kinetochore and pericentric cohesin complexes found in these regions (Vincenten *et al.* 2015; Nambiar and Smith 2018; Kuhl and Vader 2019). In addition, the centromeres of *O. polymorpha* contain repetitive LTR and Ty-like retrotransposable elements. These elements have 15-fold suppression of meiotic recombination on average compared to the rest of the genome in *S. cerevisiae* (Pan *et al.* 2011), although there is substantial variation in rates of double-strand break formation across specific Ty elements in this species (Sasaki *et al.* 2013). These features suggest that the low nucleotide sequence diversity at centromeres and across the *MAT* region in the *O. polymorpha* species complex may be the result of the low levels of recombination at these features. Rates of recombination and nucleotide diversity are hypothesized to be positively correlated due to background selection (Kaiser and Charlesworth 2009; Charlesworth and Campos 2014). Background selection can lead to Hill-Robertson interference when neutral variants are purged due to their linkage to deleterious mutations. The evidence for the relationship between recombination and nucleotide diversity varies across species of plants, animals, and fungi (Cutter and Payseur 2013), and has been shown to be correlated in *Sch. pombe* (Jeffares *et al.* 2015) and to have a weak correlation in *S. cerevisiae* (Cutter and Moses 2011; Cutter and Payseur 2013). In the methylotrophic yeast *K. phaffii*, which also uses flip/flop mating-type switching, meiotic recombination rates were 3.5X lower genome-wide than in *S. cerevisiae*, and nucleotide diversity was lower in the 150–200 kb surrounding centromeres (Braun-Galleani *et al.* 2019). In *K. phaffii*, the invertible *MAT* region is much larger than in *O. polymorpha* (138 kb) and contains a centromere. Although there was no evidence for meiotic recombination in this region in *K. phaffii*, high nucleotide diversity was observed in contrast to the other centromeres.

In “pseudo-homothallic” fungal species, linkage of the *MAT* loci to a low or no recombination region like a centromere is thought to ensure that mating types will segregate during meiosis I (Hood and Antonovics 2004; Knop 2006; Ellison *et al.* 2011). A similar logic might apply in this case, in which recombination would not only potentially prevent proper segregation of mating types in meiosis, but could result in gross structural rearrangements in the genome for a diploid that has a heterozygous inversion. The proximity of the *MAT* region to a centromere may therefore reduce the likelihood of recombination occurring in this region. In the case of *K. phaffii*, an additional set of inverted repeats within the invertible region may allow for recombination events to occur that reestablish collinearity in a diploid to allow for meiotic recombination to occur in the region (Hanson *et al.* 2014).

### CBS1977 is an interspecies diploid hybrid between *O. polymorpha* and *O. parapolymorpha*

The short-read genome assembly for CBS1977 was nearly twice the length of the other 46 isolates (Table 2), and blastn analysis of several contigs suggested that it is an interspecies diploid hybrid that resulted from a cross between *O. polymorpha* and *O. parapolymorpha*. Interspecies hybridization has played a critical role in the evolution of yeasts (Marcet-Houben and Gabaldón 2015; Gabaldón 2020) and has been demonstrated to facilitate adaption and generate biodiversity (Smukowski Heil *et al.* 2017; Tusso *et al.* 2019; Zhang *et al.* 2020). Yeast interspecies hybrids have been observed frequently in isolates from anthropogenic environments, such as industrial, agricultural, or clinical samples (Louis *et al.* 2012; Hittinger 2013; Pryszcz *et al.* 2014, 2015; Wendland 2014; Hagen *et al.* 2015; Schröder *et al.* 2016; Ortiz-Merino *et al.* 2017, 2018; Braun-Galleani *et al.* 2018; Lopandic 2018; Mixão and Gabaldón 2018; Piombo *et al.* 2018; Smukowski Heil *et al.* 2018; Samarasinghe *et al.* 2020), which may be attributed to increased stress tolerance for these isolates due to their heterozygosity. CBS1977 is potentially an example of this, as it was originally isolated from milk from a cow with mastitis (Table 1).

The high levels of heterozygosity in the CBS1977 genome reduced the quality of the short-read genome assembly (N50 = 30.1 kb; Table 2). To improve the assembly and examine the structure of the CBS1977 genome in more detail, we performed long-read MinION sequencing, which resulted in an assembly of 59 contigs with a much larger N50 of 1.26 Mb. To examine the contributions of each parent to the hybrid genome sequence, we performed a sliding window blastn analysis of the hybrid genome contigs from both the long-read and short-read assemblies against the two reference genomes for *O. polymorpha* (Riley *et al.* 2016) and *O. parapolymorpha* (Ravin *et al.* 2013).

**Figure 9:**
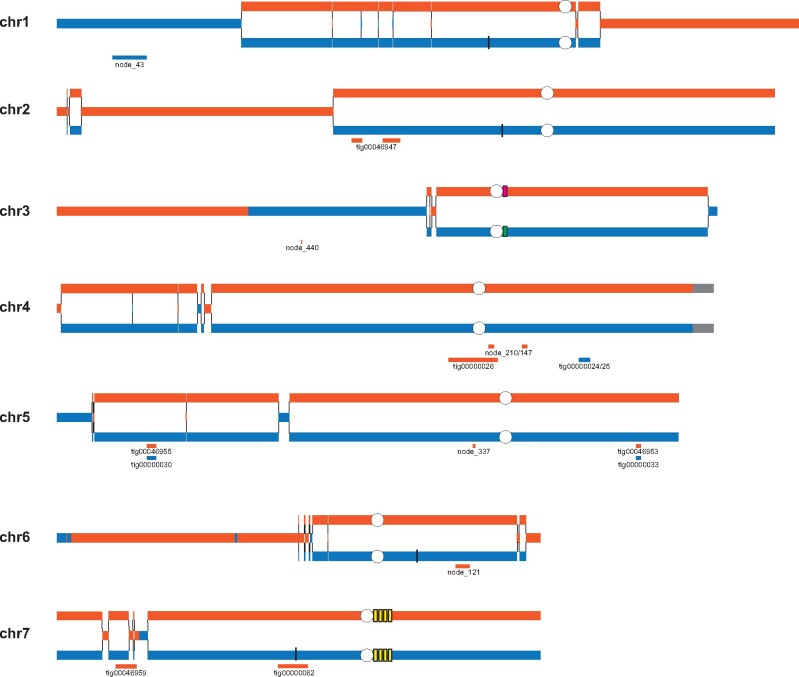
Inferred Genome Structure for Interspecies Diploid Hybrid CBS1977. Nucleotide identity for hybrid genome was determined by BLAST analysis of 1 kb sliding windows across the CBS1977 genome assembly against the *O. polymorpha* NCYC 495 and *O. parapolymorpha* DL-1 reference genome sequences. Regions that most closely match the *O. polymorpha* and *O. parapolymorpha* parental genomes are indicated in blue and orange, respectively. The right telomere of chromosome 4 could not be assigned due to high sequence identity to both parental genomes and is indicated in gray. Centromeric regions are denoted by white circles, ∼1 kb genomic repeat sequences found on NCYC 495 chromosomes 1, 2, 6, and 7 are denoted by a black line, MATa and MATα loci on chromosome 3 are denoted by green and pink boxes, respectively, and the ribosomal DNA locus on chromosome 7 is denoted by yellow boxes. Regions of the genome that contained more than one contig in either the MinION or Illumina assemblies that matched the same parental genome are indicated below the chromosome, and the name of the contigs are indicated.

Both the short-read assembly (Illumina/SPAdes) and the long-read assembly (MinION/canu, with a pilon correction step) indicated that large sections of the CBS1977 genome are heterozygous because DNA from both the *O. polymorpha* parent and the *O. parapolymorpha* parent was retained (Figure 9). However, the two assemblies differed significantly in the amount of the genome that was estimated to be heterozygous. The long-read assembly contained 3.1 Mb of heterozygous sequence, with the remaining 5.7 Mb of the genome assembly appearing to be homozygous (Table 4). However, when the latter regions were compared to the short-read assembly, an additional 2.8 Mb of the genome was found to be heterozygous (Table 4). Thus, “homozygous” regions totaling 2.8 Mb in the long-read assembly corresponded to “heterozygous” regions totaling 2 × 2.8 Mb in the short-read assembly. Since the short-read contigs in these regions matched the reference genome sequences of the two parental species, we believe that these regions are in fact heterozygous, and therefore that the difference between the two assemblies is due to over-aggressive “collapsing” of the heterozygous regions into single contigs by the canu assembler. Figure 9 shows the parental contributions that we infer from a combined analysis of the two assemblies. We also found a few regions of the genome that were represented by additional (third) long-read assembly contigs, suggesting locations where duplications may have occurred. These additional contigs are indicated in Figure 9. The mitochondrial genome of CBS1977 comes from *O. parapolymorpha.*

**Table 4:** Summary of homozygous and heterozygous composition for the interspecies diploid hybrid isolate CBS1977

Chromosome	BLAST hit length (kb)	**MinION heterozygous (kb)** *^a^*	Illumina uniquely heterozygous (kb)^*b*^	Combined heterozygous (kb)^*c*^	Homozygous opol parent (kb)^*d*^	Homozygous opar parent (kb)^*e*^	% LOH
1	1507	153	573	2	369	410	51.69
2	1565	756	276	0	0	536	34.25
3	1339	441	106	9	380	404	58.55
4	1243	493	720	0	8	22	2.41
5	1263	555	519	2	188	2	15.04
6	981	232	195	8	37	509	55.66
7	985	482	426	0	42	33	7.63
Total	8,883	3,112	2,815	21	1,024	1,916	33.08

a Total length of heterozygous regions supported by MinION assembly.

b Total length of heterozygous regions supported only by Illumina assembly (homozygous in MinION assembly).

c Total length of heterozygous regions supported by one Illumina contig and one MinION contig or scaffold.

d Total length of homozygous regions that have higher sequence identity to Opol parental genome.

e Total length of homozygous regions that have higher sequence identity to Opar parental genome.

After determining the heterozygous regions from the combination of long-read and short-read assemblies, we found loss of heterozygosity (LOH) has occurred for 33% of the genome in CBS1977 (Table 4). The interspecies hybrid likely formed through a mating event between two haploid cells, based on the presence on chromosome 3 of one *MAT* locus contributed by each parental species (Figure 9, Supplementary Figure S4). The homozygous regions of the genome are derived from both the *O. polymorpha* and *O. parapolymorpha* parental genomes (Table 4), suggesting that the LOH has not resulted from backcrossing of the hybrid to a specific parent. Heterozygosity has been maintained for the centromeric regions of each chromosome, which includes the ribosomal DNA locus adjacent to the centromere on chromosome 7 (Figure 9), so CBS1977 has retained both of the parental rDNA sequences. The maintenance of heterozygosity in centromeric regions may indicate a general suppression of recombination at these sites, consistent with observed recombination patterns in yeasts (Peter *et al.* 2018; Tattini *et al.* 2019).

Genome stabilization through LOH has frequently been observed following hybridization events (Morales and Dujon 2012), and has been associated with adaptation (Smukowski Heil *et al.* 2017; Zhang *et al.* 2020). LOH results from homologous recombination that resolves by reciprocal genetic exchange (interhomolog crossover) or nonreciprocal genetic exchange between homologs (gene conversion or break-induced replication) (Symington *et al.* 2014). In *O. polymorpha* and *O. parapolymorpha*, the sexual processes of mating and sporulation are induced by the same environmental conditions (nitrogen starvation), resulting in transient diploids that readily enter meiosis to return to a haploid state (Hanson *et al.* 2014; Maekawa and Kaneko 2014). The sustained diploid state of the hybrid suggests that it cannot sporulate, despite the ability of a laboratory-created interspecies hybrid between *O. polymorpha* and *O. parapolymorpha* to undergo meiosis (Hanson *et al.* 2017). Crossover events are rare in mitotic recombination, in which synthesis-dependent strand annealing (SDSA) is a more common mechanism for double-strand break resolution (Symington *et al.* 2014). Small interspersed regions of LOH, which are found throughout the genome of CBS1977 (Figure 9), may be accounted for by SDSA. However, much of the LOH in the genome has occurred in long stretches at the ends of chromosomes (Figure 9). This pattern is more readily explained by break-induced replication (BIR), which is a replication-dependent nonreciprocal genetic exchange that occurs when only one end of a DSB is used for homologous recombination (Kramara *et al.* 2018). An experimental evolution study with *S. cerevisiae* × *S. paradoxus* hybrids showed that gene conversion events leading to LOH were reduced relative to intraspecies hybrids, which may be explained by reduced recombination due to sequence differences between the parental genomes. BIR occurs more frequently under stressful conditions (Kramara *et al.* 2018), as well as when only one side of a DSB has homology with a repair template (Malkova *et al.* 2005; Symington *et al.* 2014), which may explain why BIR is prevalent in hybrid genomes, which occur more frequently in conditions requiring stress-tolerance and where sequence divergence between homologous chromosomes may make recombination less efficient.

## Conclusions

Our study is a first examination of the population genomics of the *O. polymorpha* species complex. Using phylogenomics, we have established the relationships among the four species in the complex. We surveyed the genetic variation within and between species by comparing the genome sequences of 47 isolates. We found evidence for structural rearrangements in *O. polymorpha*, *O. angusta*, and *O. haglerorum*, and identified one isolate as an interspecies hybrid between *O. polymorpha* and *O. parapolymorpha* that formed through haploid mating and has since undergone loss of heterozygosity. These data will provide a useful resource for the continued use of *O. polymorpha* as a model system in genetics, cell biology, and recombinant protein production.

## Supplementary Material

jkab211_Supplementary_Data
