# Supplementary material for: Genomic diversity, chromosomal rearrangements, and interspecies hybridization in the Ogataea polymorpha species complex
Source: G3 (Bethesda). 2021 Jun 18;11(8):jkab211. doi: 10.1093/g3journal/jkab211 (PMC8496258; doi:10.1093/g3journal/jkab211)
Supplement: jkab211_Supplementary_Data [file jkab211_supplementary_data.zip › jkab211-suppl_data/GENETICS-G3-2021-402608-s01.docx]

**Figure S1. Phylogenomic analysis of *Ogataea polymorpha* species complex isolates.** Supermatrix phylogeny of *O. polymorpha* species complex isolates with 24 *Ogataea* species and *P. kudriavzevii* derived from 1,148 BUSCO families giving an alignment 632,568 amino acids in length. Maximum Likelihood phylogeny was reconstructed with IQ-TREE implementing the JTT+F+R5 model. Bootstrap support values are indicated at all nodes.

**Figure S2. BUSCO analysis of *Ogataea* genomes.**

**Figure S3. Copy Number Variants (CNVs) in *Ogataea polymorpha* species complex.** Heatmap showing duplications (yellow) and deletions (blue) greater than 50 base pairs in length identified in the genomes of isolates compared to a reference genome for each species (*O. polymorpha* NCYC495, *O. parapolymorpha* DL-1, *O. angusta* Oang9, and *O. haglerorum* Ohag10). Location of each CNV relative to the *O. polymorpha* NCYC495 genome assembly is indicated at the top (Chr. 1-7), and the loci impacted by the CNV are listed at the bottom.

**Figure S4. Structure of MAT region in Interspecies Diploid Hybrid Isolate CBS1977.** Schematic of chromosome 3 centromere, 19 kb MAT region content, and 2 kb inverted repeats (indicated in cyan) drawn to scale. The genes specifying mating-type a are shown in green, and those specifying mating-type a are shown in pink. Genes that share nucleotide identity with *O. polymorpha* NCYC495 and *O. parapolymorpha* DL-1 genomes are indicated in dark blue and orange, respectively. The centromeric regions on each chromosome (marked by the purple rectangles) contain long terminal repeat sequences (denoted by black boxes).

**Table S1.** Summary of *O. parapolymorpha* genetic variation by scaffold.

**Table S2.** Summary of *O. haglerorum* genetic variation across 22 largest contigs.

**Table S3.** Summary of *O. angusta* genetic variation across 15 largest contigs.

**Table S4.** Summary of *O. polymorpha* genetic variation by chromosome.

**Table S5.** Summary of genetic variation in chromosomal regions in *Ogataea*.
