# Supplementary figures and images for: Genomic diversity, chromosomal rearrangements, and interspecies hybridization in the Ogataea polymorpha species complex
Source: G3 (Bethesda). 2021 Jun 18;11(8):jkab211. doi: 10.1093/g3journal/jkab211 (PMC8496258; doi:10.1093/g3journal/jkab211)

Tree scale: 0.1

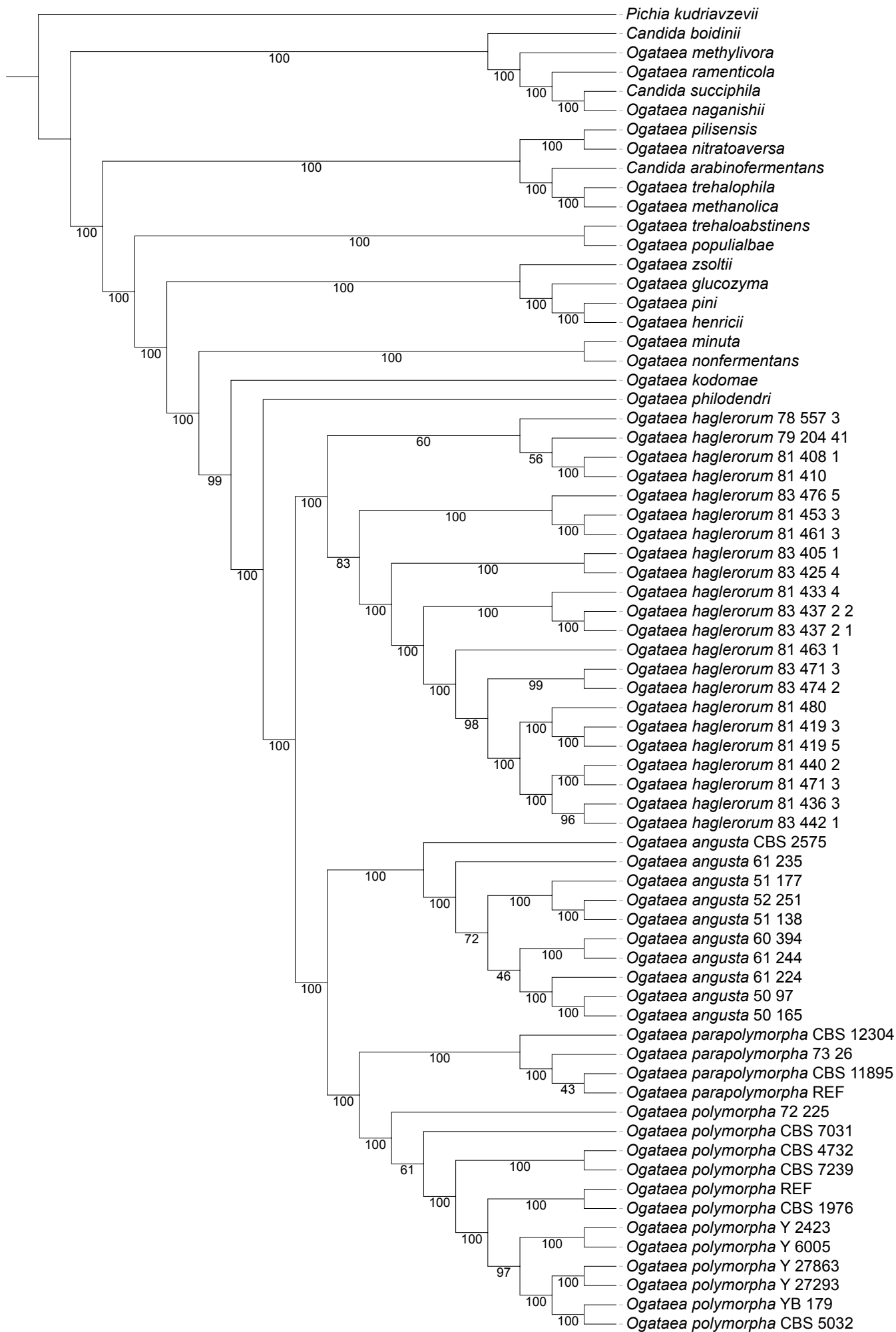

Supplement: jkab211_Supplementary_Data [file jkab211_supplementary_data.zip › jkab211-suppl_data/GENETICS-G3-2021-402608-s02.pdf]

BUSCO Assessment Results

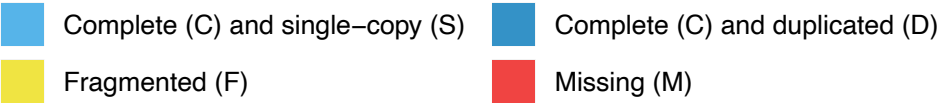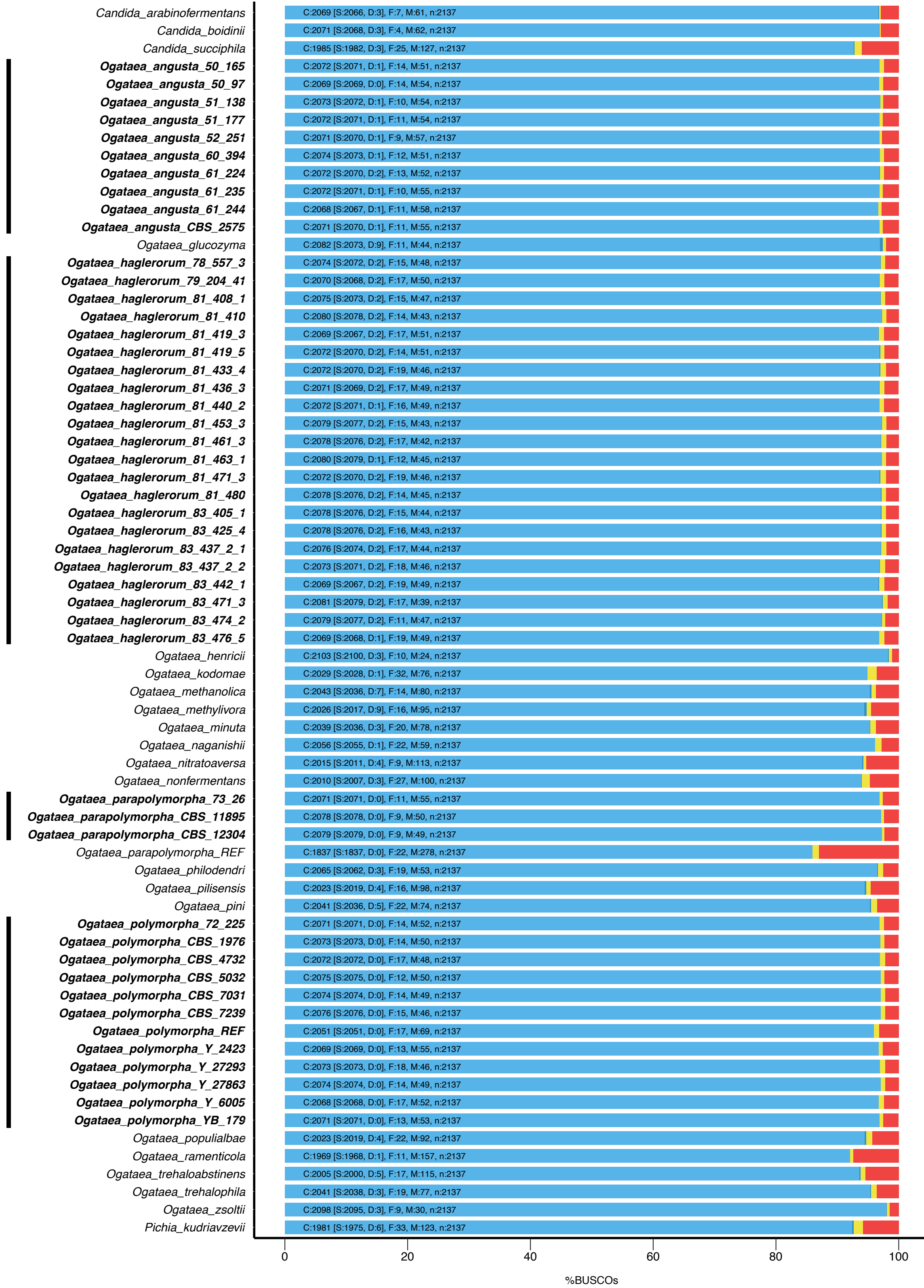

Supplement: jkab211_Supplementary_Data [file jkab211_supplementary_data.zip › jkab211-suppl_data/GENETICS-G3-2021-402608-s03.pdf]

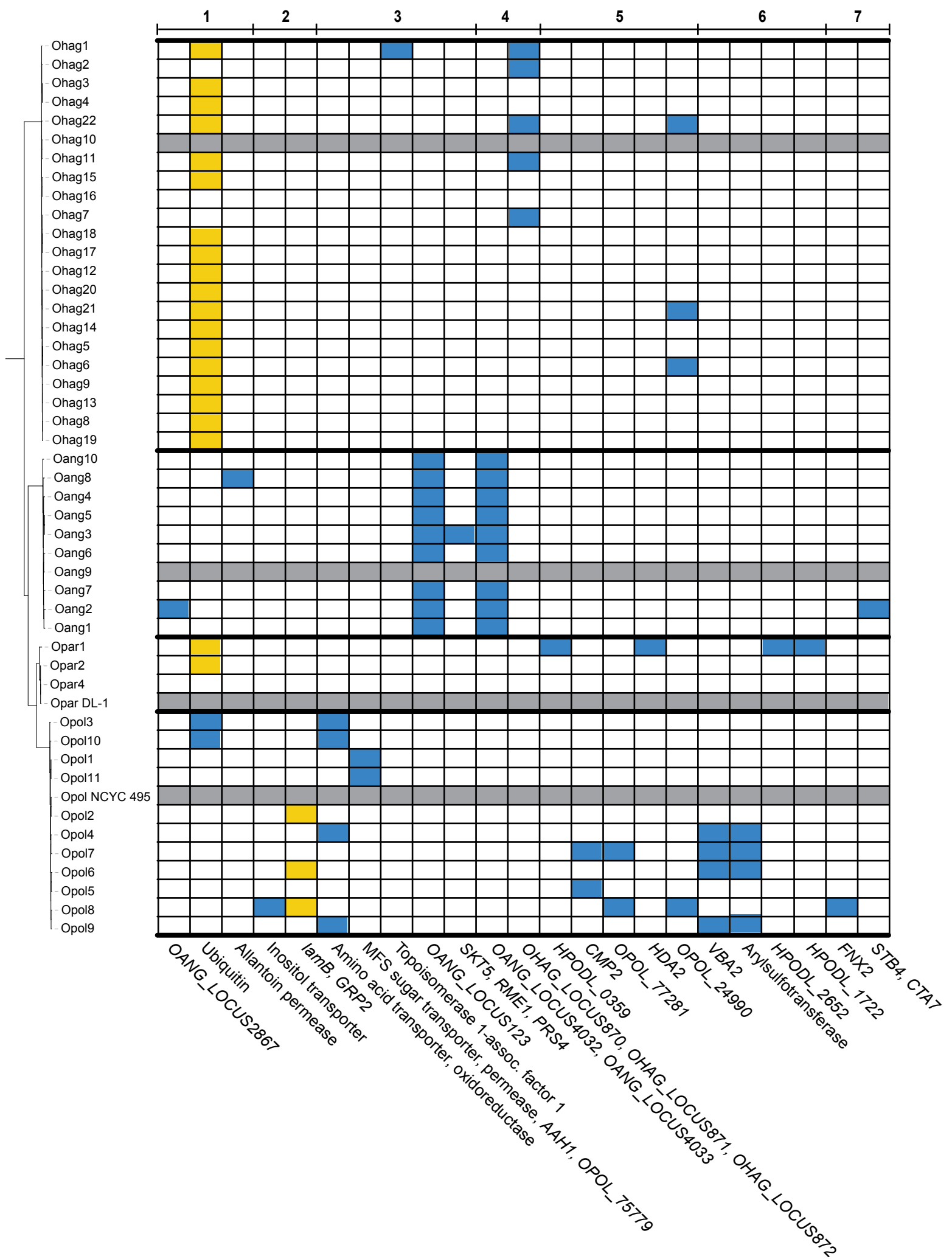

Supplement: jkab211_Supplementary_Data [file jkab211_supplementary_data.zip › jkab211-suppl_data/GENETICS-G3-2021-402608-s04.pdf]

tig00000014

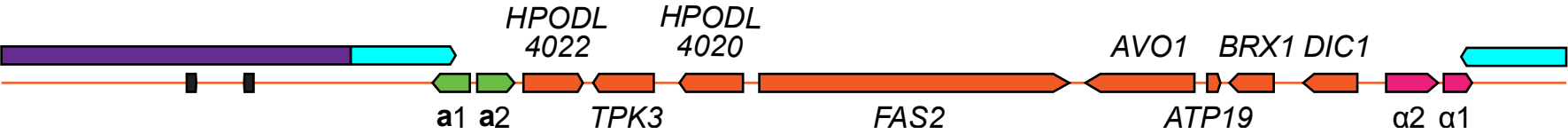

tig00000016

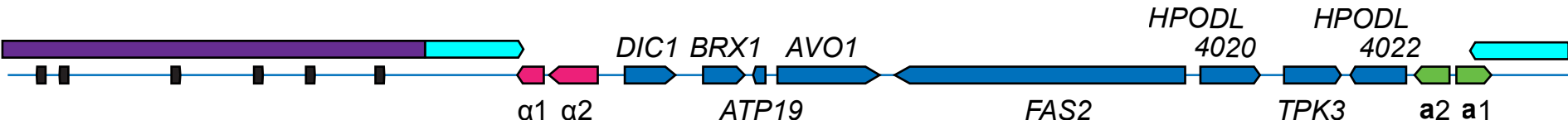

Supplement: jkab211_Supplementary_Data [file jkab211_supplementary_data.zip › jkab211-suppl_data/GENETICS-G3-2021-402608-s05.pdf]
